# The increasing need for systematic reviews of prognosis studies: strategies to facilitate review production and improve quality of primary research

**DOI:** 10.1186/s41512-019-0049-6

**Published:** 2019-01-23

**Authors:** Johanna A. A. G. Damen, Lotty Hooft

**Affiliations:** 1 Cochrane Netherlands, University Medical Center Utrecht, Utrecht University, Utrecht, The Netherlands; 2 Julius Center for Health Sciences and Primary Care, University Medical Center Utrecht, Utrecht University, Utrecht, 3508 GA The Netherlands

**Keywords:** Prognosis, Prediction, Systematic review, Meta-analysis

## Abstract

Personalized, precision, and risk-based medicine are becoming increasingly important in medicine. These involve the use of information about the prognosis of a patient, to make individualized treatment decisions. This has led to an accumulating amount of literature available on prognosis studies. To summarize and evaluate this information overload, high-quality systematic reviews are essential, additionally helping us to facilitate interpretation and usability of prognosis study findings and to identify gaps in literature. Four types of prognosis studies can be identified: overall prognosis, prognostic factors, prognostic models, and predictors of treatment effect. Methodologists have focussed on developing methods and tools for every step of a systematic review for reviews of all four types of prognosis studies, from formulating the review question and writing a protocol to searching for studies, assessing risk of bias, meta-analysing results, and interpretation of results. The growing attention for prognosis research has led to the introduction of the Cochrane Prognosis Methods Group (PMG). Since 2016, reviews of prognosis studies are formally implemented within Cochrane. With these recent methodological developments and tools, and the implementation within Cochrane, it becomes increasingly feasible to perform high-quality reviews of prognosis studies that will have an impact on clinical practice.

Clinical practice roughly consists of diagnosis, prognosis, and treatment. Treatment selection used to be driven mostly by the diagnosis that was made, but prognosis has become increasingly important to inform evidence-based decisions about healthcare. Clinical trials mainly focused on estimating a single overall effect, while decisions are being made on an individual level. Some patients benefit more (or less) than average; therefore, identifying those subgroups with different effects of an intervention has become very important to guide evidence-informed decision making. This personalized, precision, and risk-based medicine all involves the use of prognostic and predictive characteristics to make individualized treatment decisions for patients. The shift to personalized medicine has led to an accumulating amount of evidence available from prognosis studies. Reviews of prognosis studies make this information overload informative and usable. They summarize and evaluate the available evidence and guide the interpretation of results, in order to facilitate optimal use of all existing evidence. Reviews of high quality provide trusted evidence for stakeholders, like clinicians and guideline developers, to help them deciding which prognostic model or factor to use in clinical practice or implement in evidence-based guidelines. In addition, reviews identify gaps and redundant or unnecessary studies in the scientific literature, highlight flaws in conduct and reporting of primary studies, and identify and indicate which further studies are needed [1–5]. Therefore, reviews should serve as the essential starting point for clinical researchers of primary studies when designing a new prognosis study. The aim of this editorial is to provide an overview of the improvements in methods to perform systematic reviews of prognosis studies and freely available tools and templates. In addition, we want to raise awareness amongst clinical researchers of primary prognosis studies that those reviews and tools (e.g. reporting guidelines) are essential to use when a new study is designed, conducted, and reported. Our ultimate goal is to facilitate the production of only necessary, highly relevant, and unbiased reviews, which provide an overview of high-quality and useful primary prognosis studies.

Primary prognosis studies are presented as four types in the PROGnosis RESearch Strategy (PROGRESS) partnership series [6–9]: overall prognosis, prognostic factors, prognostic models, and predictors of treatment effect (also known as predictive factors, or treatment selection factors). Studies on (1) overall prognosis give insight in the occurrence of certain outcomes in a certain time frame, of a group of individuals with a certain health condition (not necessarily a disease). An example can be to study the overall survival in women with ovarian cancer. Studies on (2) prognostic factors identify variables that are prognostic for a certain outcome in a certain individual within a given timeframe, e.g. the prognostic value of c-reactive protein in predicting the 10-year risk of cardiovascular disease. Prognostic model studies (3) combine prognostic factors in a single model to make personalized predictions for individuals with a certain health condition and study the development and transportability or generalizability of a model to other populations. For example, the Pneumonia Severity Index (PSI) combines predictors like age, comorbidities, physical findings, and laboratory findings to estimate the 30-day mortality rate in patients with community-acquired pneumonia [10]. A validation study of the PSI showed that this model is not suitable to use in people with bacteraemic pneumococcal pneumonia [11]. Studies on predictors of treatment effect (4) aim to identify individuals’ factors that are associated with the effectiveness of a certain treatment, e.g. the presence of the oncogene HER2/neu is predictive of the effectivity of the monoclonal antibody trastuzumab for treating breast cancer [12]. An additional primary study type is where several predictors of treatment effect are combined, to form a predictive model that predicts treatment effect. A model like this can be used to select individuals that benefit most from a certain treatment.

All types of primary prognosis studies can be summarized, evaluated, and interpreted in different types of systematic reviews, following the broad range of aims and objectives of the included prognosis studies. Reviews are, for example, helpful to give an overview of all available prognostic factors or models (e.g. to identify all factors or models for the prediction of heart failure in patients with type 2 diabetes mellitus), to study the prognostic value of a certain externally validated prognostic model (e.g. the predictive (prognostic) performance of the Revised Cardiac Risk Score for cardiac outcomes after noncardiac surgery [13]), or the added value of one or more predictors on top of an existing model (e.g. the added value of coronary artery calcification to a model for cardiovascular disease prediction [14]). As usually prognosis studies suffer from extensive heterogeneity in selected populations and the measurement and definition of predictors and outcomes, most systematic reviews also aim to identify sources of this heterogeneity.

Methodological guidance for most steps of conducting a systematic review is currently developed. Amongst others, to facilitate searching and reduce the number of references to be screened, methodological search filters are available [15–18], and increasingly, data mining tools (like [19]) are being developed that can identify discriminative words to narrow down search results. To facilitate framing the review question, and data extraction and critical appraisal of prognostic model studies, the Critical Appraisal and Data Extraction for Systematic Reviews of Prediction Modelling Studies (CHARMS) checklist has been developed (though it can also be adopted to other types of prognosis studies) [20]. Risk of bias assessment of included studies can be challenging, as evidence on the influence of design choices on the performance of a model is limited. However, tools for risk of bias assessment for both prognostic factor studies (QUIPS) and prediction models (PROBAST) are available [21, 22]. It can also be challenging to perform a quantitative synthesis (meta-analysis) of the results of prognosis studies due to heterogeneity in selected populations, measurement and definitions of predictors and outcomes, and reporting of performance measures. Methods to deal with these issues in reviews of prognostic model and prognostic factor studies have been described [23–25]. Further, we are working on guidance for presenting and interpreting the results of systematic reviews and guidance for reporting systematic reviews. For primary prognosis studies, the transparent reporting of a multivariable prediction model for individual prognosis or diagnosis (TRIPOD) statement has been published in 2015, to guide completeness of reporting of essential elements of primary prediction studies [26, 27]. Hopefully, this will improve the reporting and therefore lead to more informative systematic reviews.

The growing attention for prognosis research and the increasing emphasis on the importance of prognostic information in clinical practice have led to the introduction of the Cochrane Prognosis Methods Group (PMG) in 2007 [28]. Over the years, a growing group of experts in the field of primary prognosis studies and evidence synthesis have joined this group to work together and develop tools and guidance necessary for facilitating reviews of prognosis studies. Since 2016, reviews of prognosis studies are formally adopted and implemented within Cochrane (via the Cochrane PMG [29]). The first two Cochrane reviews were published in 2018 [30, 31], ten protocols are published in the Cochrane Library, and five titles have been registered. The implementation within Cochrane comes together with the development of tools and templates for conducting a review of prognosis studies. Trainings and webinars are organized by the Cochrane PMG, aiming to give researchers sufficient skills on how to use the tools and templates and up-to-date knowledge on performing a systematic review of prognosis studies (see [29] for available tools and templates). All tools, templates, and methods developed by researchers involved with Cochrane are also available for authors writing a non-Cochrane review.

In summary, systematic reviews are urgently needed to summarize the growing amount of prognostic evidence, to evaluate the available evidence and guide the interpretation of results, in order to facilitate optimal use of existing evidence for medical practice and policy making. With the recent methodological developments and tools for systematic reviews of prognosis studies, it becomes increasingly feasible to perform these reviews. With the implementation within Cochrane, it is ensured that reviews of high quality will be produced that will have an impact on clinical practice.

## Abbreviations

CHARMS: Critical Appraisal and Data Extraction for Systematic Reviews of Prediction Modelling Studies
PMG: Prognosis Methods Group
TRIPOD: Transparent reporting of a multivariable prediction model for individual prognosis or diagnosis
